# Transcriptions of *ACO* and *ACS* genes are involved in nitrate-dependent root growth of maize seedlings

**DOI:** 10.3389/fpls.2025.1566213

**Published:** 2025-05-02

**Authors:** Guoqi Yao, Zhenwei Yan, Shijun Ma, Xia Liu, Juan Shan, Bing Cao, Xiaole Chen, Bingying Leng, Chunhua Mu

**Affiliations:** ^1^ Maize Research Institute, Shandong Academy of Agricultural Sciences, Jinan, China; ^2^ Plant Protection Research Institute, Rizhao Academy of Agricultural Sciences, Rizhao, China

**Keywords:** maize, nitrate, ethylene synthesis pathway, ACO, ACS, root growth

## Abstract

Improving nitrogen use efficiency (NUE) is one of the major objectives for crop breeding. As nitrate signaling plays pivotal roles in nitrogen use of plants, factors in this pathway might be valuable for improving the NUE of maize. In this research, we performed Gene Set Enrichment Analysis (GSEA) of maize transcriptomes in response to nitrate and found that the ethylene action pathway might participate in nitrate signaling. Through a modified reciprocal best hit approach, we obtained 16 maize aminocyclopropane-1-carboxylic acid (ACC) oxidase (ACO) and four ACC synthase (ACS) homologs in maize genome. *In silico* analyses and the reverse transcription quantitative PCR assays demonstrated that *ZmACCO7*, *ZmACCO5*, *ZmACCO15*, *ZmACCO35*, and *ZmACCO31* are the top five highly expressed *ACO* genes, and *ZmACS1* is the most highly expressed *ACS* gene in the primary and seminal roots of maize. We discovered that *ACO* and *ACS* genes have different regulatory modes in response to nitrate provision. Some *ACO* genes, which are mainly expressed in root regions far from the root tip like *ZmACCO7*, are repressed by nitrate, while the others, which are mainly expressed in root regions near the root tip like *ZmACCO5*, are induced by nitrate. *ZmACS1*, which has more uniform expression across maize roots, is induced in root regions near the root tip and repressed in regions far from the root tip. A phenotypic analysis indicated that upregulation of *ACO* and *ACS* genes by nitrate is linked to repression of axial root elongation by nitrate while the downregulation of these genes is associated with the promotion of growth of lateral roots of the axial roots. In addition, differences in regulation of *ACO* and *ACS* genes by nitrate were observed between genotypes, which is related to the differences in the responses of their primary root growth to nitrate. These results suggested that the ethylene synthesis pathway is involved in the responses of maize roots to nitrate, which is associated with the remodeling of maize root architecture by nitrate.

## 1 Introduction

Nitrogen fertilizer is one of the major investments for crop production due to the high amount of requirement of nitrogen of plants and limited nitrogen available in the soil. Moreover, application of fertilizer leads to the eutrophication of aquatic ecosystems (Raun and Johnson, 1999), and the production of fertilizer also causes air pollution. Therefore, improving nitrogen use efficiency (NUE) becomes one of the major objectives of crop breeding programs. However, as a complex trait, it is a challenge at present. Since nitrate is the predominant nitrogen form available to plants in aerated soil (Dechorgnat et al., 2011), nitrate uptake, transport, metabolism, and their regulation are the key processes to enhance the NUE of arable crops like maize.

The root architecture and the activity of nitrate transporters of root cells play critical roles in the NUE of crops as roots are the main organs for plants to absorb nitrate from soils (Aluko et al., 2023; Hawkesford and Griffiths, 2019). A comparison between hybrids from different eras showed that the remodeled root architectures in the breeding processes enhanced the NUE of maize (Li et al., 2024). Genes in the pathway of auxin signaling were found to be key elements in regulating maize root development (Hochholdinger et al., 2018). A total of 81 high-priority candidate genes associated with root angle were identified by integrating genome-wide association analysis with co-expression network analysis (Ren et al., 2022). Research in rice demonstrated that *NRT1.1B-indica* allele improved the grain yield and NUE when introduced into varieties without this allele (Hu et al., 2015). In maize, 78 *Nitrate Transporter1/Peptide Transporter Family* (*NPF*), seven *Nitrate Transporters2* (*NRT2*), and two *NRT3* genes were reported (Jia et al., 2023). Transcriptional factors involved in the nitrate signaling were also valuable in improving the NUE of crops. For example, *OsTCP19* is associated with a high tillering response to nitrogen (Liu et al., 2021).

Ethylene is a plant hormone regulating numerous physiological and morphological responses of plants by interacting with other signaling molecules (Khan et al., 2015). Generally, ethylene is regarded as a stress hormone (Wang et al., 2002). Suboptimal N status, either deficiency or excess, acts as a stress to stimulate ethylene biosynthesis and signaling to remodel root architectures, nitrogen uptake, and translocation (Ma et al., 2023). High nitrate concentration (10 mmol/L) induces ethylene synthesis in the root of *Arabidopsis* (Tian et al., 2009). Low nitrate treatment also induces a rapid burst of ethylene production (Zheng et al., 2013). Research in *Arabidopsis* showed that ethylene interacts with cytokinin through ethylene receptor ETR1 to control primary root growth (Zdarska et al., 2019) and modulates the transcription of auxin carrier genes to inhibit lateral root formation and elongation (Lewis et al., 2011). Ethylene also regulates the growth of root hairs through transcriptional factor ethylene insensitive3 (EIN3) (Xiao et al., 2021). In addition, the production of ethylene represses the expression of the low-affinity nitrate transporter coding gene *NRT2.1* but upregulates *NRT1.1* expression in *Arabidopsis* (Tian et al., 2009).

The synthesis of ethylene from its general precursor S-adenosyl-l-methionine (SAM) in plants mainly involves two processes. The first step is the conversion of SAM to 1-aminocyclopropane-1-carboxylic acid (ACC), which is catalyzed by ACC synthase (ACS). The second step is the production of ethylene from ACC, which is catalyzed by ACC oxidase (ACO) (Wang et al., 2002). The pathway of ethylene production is precisely tuned at both transcriptional and post-translational levels (Houben and Van de Poel, 2019; Pattyn et al., 2021). The conversion of SAM to ACC was considered as the rate-limiting step in ethylene biosynthesis (Tsuchisaka et al., 2009). Evidence also showed that the activities of ACOs are the rate-limiting factors during some processes (Houben and Van de Poel, 2019). Maize *ZmACS7* over-expressors displayed accelerated leaf senescence in response to N deficiency and improved NUE (Xing et al., 2024). However, how ethylene synthesis and signaling pathways are involved in the nitrate-regulated plant growth in maize remains poorly understood.

Here we reported that public data mining indicated that the ethylene action pathway is disturbed by nitrate in maize roots, and experiments showed that transcriptions of *ACO* and *ACS* genes are regulated by nitrate with different modes in maize seedling roots, which is related to nitrate-modulated root growth.

## 2 Materials and methods

### 2.1 Plant materials and growth condition

The inbred lines B73 and Z58 were used in this study. Z58 (Zheng58) is a parent of Zhengdan958, a widely planted hybrid in North China. Plant seeds were sterilized using 1:20 dilution of sodium hypochlorite for 15 min, washed six times with deionized water, and germinated on a wet towel for 2 days at 25°C. The seedlings were grown in paper rolls containing 0.1 mmol/L CaCl_2_ for 3 days at 25°C and then fixed to boxes full of 0.1 mmol/L CaCl_2_ solution to grow in a chamber with controlled climate conditions (25°C) for 1 day. Subsequently, the seedlings were supplied with nutrient solution containing different concentrations of nitrogen to grow after their endosperms were removed.

The base nutrient solution contained 0.75 mmol/L K_2_SO_4_, 0.1 mmol/L KCl, 0.25 mmol/L KH_2_PO_4_, 0.65 mmol/L MgSO_4_, 0.2 mmol/L EDTA-Fe, 1.0 μmol/L H_3_BO_3_, 1.0 μmol/L MnSO_4_, 1.0 μmol/L ZnSO_4_, 0.1 μmol/L CuSO_4_, and 0.005 μmol/L (NH_4_)_6_Mo_7_O_24_. For high nitrogen solution (HN), Ca(NO_3_)_2_ was added into the base nutrient solution at a concentration of 2.0 mmol/L, and for low nitrogen solution (LN), CaCl_2_ was added at a concentration of 2.0 mmol/L. The pH of the growth solutions was adjusted to 6.0 with NaOH. For cobalt treatments, nutrient solutions containing 3.0 μmol/L CoCl_2_ were used. For hydrophonic culture, the solutions were ventilated for 20 min each 2 h by electric pumps.

For the analysis of gene expression in response to nitrate, seedlings were grown in LN for 1 day, and then the solutions were replaced with fresh LN (as the control) or HN solutions. To examine nitrate’s effects on the phenotype of plants, seedlings were grown in LN or HN for 9 days, and the solutions were renewed every 2 days.

### 2.2 RNA extraction, reverse transcription, and quantitative PCR

Seedling roots were harvested for RNA extraction. The total RNA was prepared using Aidlab’s plant RNA extraction kit RN09 (Aidlab, Beijing, China) and reverse transcribed to cDNA using Accurate Biology’s RT Kit (Accurate Biology, Changsha, China). Quantitative PCRs (qPCR) were performed with SYBR Green Real-Time PCR Master Mix (Accurate Biology, Changsha, China) using Applied Biosystems 7500 Real-Time PCR System (Thermo Fisher Scientific, Waltham, MA, USA) following the comparative CT experiment protocol of the manufacturer. The number of biological repeats were described in figures in detail. For the analysis of root-region-specific mRNA expression of genes in response to nitrate provision, 14 plant samples were bulked for RNA extraction unless otherwise stated in the text. For the mRNA expression analysis in other cases, six to seven biological replicates, each of which contained one plant sample, were performed. Two to three technical replicates were done and folypolyglutamate synthase (*FPGS*) was used as the reference gene (Manoli et al., 2012) in qPCR assays. Relative gene expression was calculated as described by Livak and Schmittgen (2001). The primers used in the research are listed in **Supplementary Table S1**.

### 2.3 Measurement of root length, number of lateral roots and plant height, and statistical analysis

Plant height, the length from the node where the coleoptile grows to the tip of the longest leaf of a seedling, was measured with a ruler. Each primary and seminal root was measured separately. After being detached from the plant at the base, an axial root with branched lateral roots was floated in a transparent plastic tray (20 cm × 15 cm) full of water and next scanned with Epson Perfection V700 Photo (EPSON, Beijing, China), followed by measuring the axial root length with a ruler. The resulting images were analyzed using the software WinRHIZO Pro 5.0 (Quebec City, Canada). The number of root tips obtained with WinRHIZO was considered as the number of lateral roots. The total lateral root length was calculated by subtracting the axial root length from the total root length derived from WinRHIZO. Each group of treatments was conducted in an independent experiment and was analyzed separately with two-tailed *t*-test in the phenotype evaluation. For seminal roots, the mean of values of a trait per plant was used for further analysis.

### 2.4 Analysis of RNA−Seq data

Transcriptomic data from PRJNA283053 and PRJNA304223 were used for Gene Set Enrichment Analysis (GSEA). *In silico* organ- or tissue-specific expression patterns of *ACS* and *ACO* genes were estimated with transcriptomic data from PRJNA217053 and PRJNA171684. These data were downloaded from https://www.ebi.ac.uk/ (accessed on January 20, 2020). Reads with poor quality were filtered with Trimmomatic V0.39 (Bolger et al., 2014). The clean data passing the quality control were evaluated with FastQC V 0.11.8 (https://www.bioinformatics.babraham.ac.uk/projects/fastqc/) and used to quantify transcripts with Salmon V1.0.0 (Patro et al., 2017) based on the gene models of Zm-B73-REFERENCE-GRAMENE-4.0 (https://www.maizegdb.org/, accessed on December 1, 2019). Gene expression changes were determined using the R package DESeq2 V1.42.1 (Love et al., 2014). GSEA (Subramanian et al., 2005) was performed by using the software GSEA V4.3.3 with Signal2Noise being selected as metric for ranking genes and gene sets being used for permutation. Gene counts calculated with transcript counts derived from Salmon analysis and then normalized with EdgeR package V3.20 (Robinson et al., 2010) were used for further GSEA. Gene sets were constructed according to the maize gene pathway annotation of MapMan (Thimm et al., 2004) based on gene models of Zm-B73-REFERENCE-GRAMENE-4.0.

### 2.5 Maize ACS and ACO homolog searching and phylogenetic analysis

A modified reciprocal best hit (RBH) (Bork et al., 1998; Tatusov et al., 1997) method was used to obtain maize ACS and ACO homologs. In brief, *Arabidopsis* ACO (Houben and Van de Poel, 2019) and ACS proteins (Yamagami et al., 2003) were used to blast against predicted maize proteins from the gene models of Zm-B73-REFERENCE-GRAMENE-4.0. Top hits, with e-value being less than 1 × 10^−20^ as well as both query and subject coverage being more than 50%, were kept for reverse blast against the *Arabidopsis* protein database (TAIR10). A maize homolog was considered as a true homolog when a positive reciprocal blast search was obtained. The canonical transcript for a maize gene and the representative transcript for an *Arabidopsis* gene were considered for further analyses where there were several transcripts for a gene. Sequences were aligned by ClustalW, and phylogenetic trees were constructed with the maximum likelihood method implemented in MEGAX V10.0.5 (Kumar et al., 2018). A total of 1,000 replicates of bootstraps were performed in the phylogenetic analyses.

## 3 Results

### 3.1 The ethylene action pathway was enriched in the root transcriptomic response to nitrate in maize

**Figure 1:**
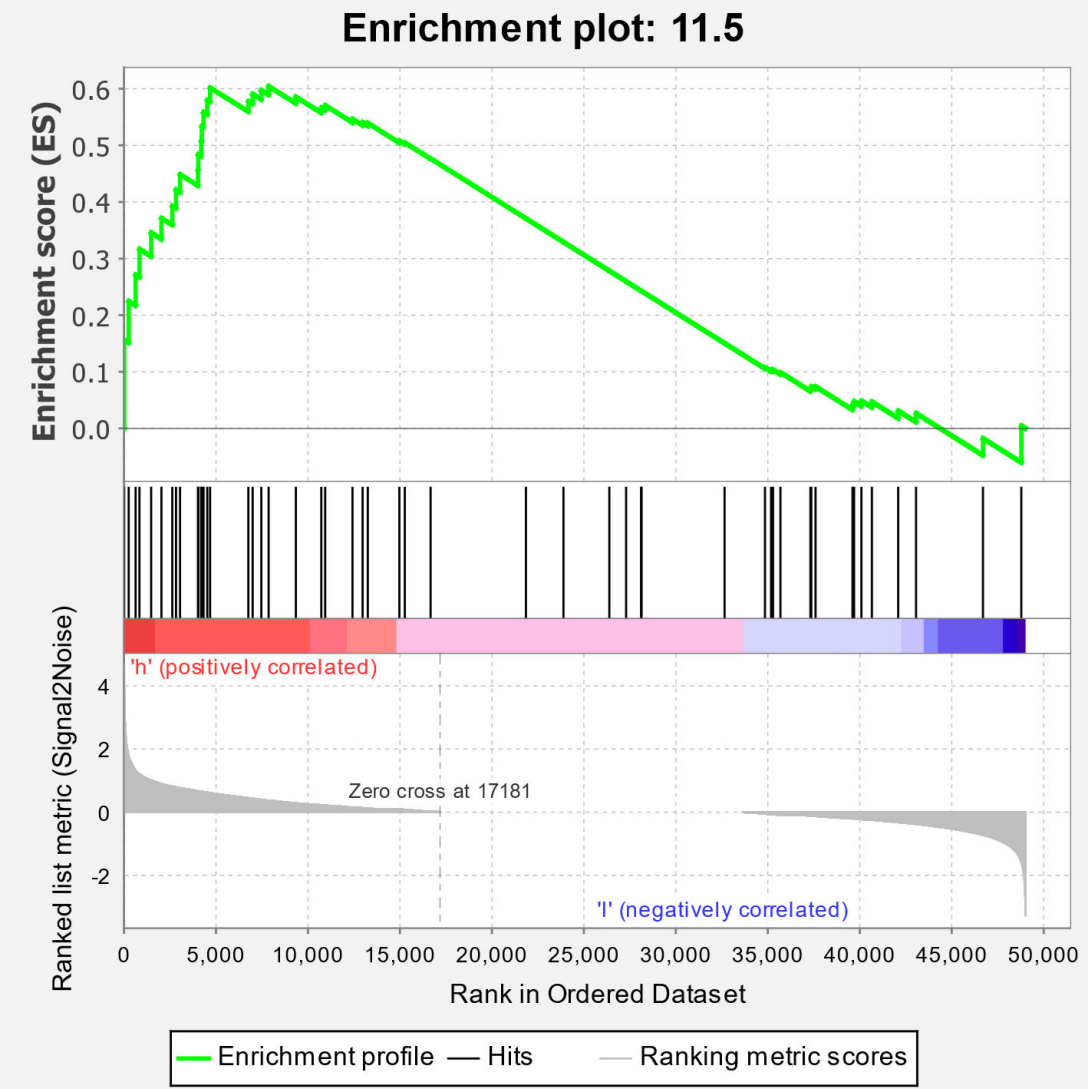
Enrichment of the ethylene action pathway (pathway 11.5 in MapMan) in maize transcriptomic responses to nitrate treatment. Clean data derived from transcriptomic data of PRJNA283053 were subjected to quantify transcripts with Salmon-SMEM to get gene counts. Enrichment scores were calculated using the software GSEA-MSigDB with edgeR-normalized gene counts used as expression data and MapMan-derived gene pathways used as gene sets.

As nitrate signaling plays pivotal roles in the use of nitrogen in plants, factors in this process might be valuable to improve the NUE of maize. Thus, we conducted the public transcriptomic data mining in order to identify key genes in nitrate signaling as candidates to enhance the NUE of maize. PRJNA283053 (Yu et al., 2015) was a project of transcriptomic assays of stele tissues extracted from the regions between 5 and 25 mm of shoot-borne roots of B73 in response to 24-h local high-nitrate stimulation. The project of PRJNA304223 included comparative transcriptome data of roots of two genotypes, the *rtcs* (*rootless concerning crown and seminal roots*) mutant and the wild type B73, in response to different concentrations of nitrate (He et al., 2016). The B73 transcriptomes in these two data sets were downloaded for analyses. GSEA is considered as a robust method to identify *a priori* defined gene set differentially affected between experiment groups (Subramanian et al., 2005). Thus, we performed GSEA of the expression data with MapMan pathway (Thimm et al., 2004) used as gene sets. In the analysis of data from PRJNA283053, we found that ethylene action (11.5 in MapMan) was the third significantly enriched pathway (**Figure 1**; **Supplementary Table S2**). A detailed examination showed that 10 were annotated as *ACO* or *ACS* genes, and five of them were significantly upregulated by high nitrate treatment among 20 genes in the core enrichment (**Table 1**). However, this result was not confirmed in the analysis of B73 transcriptomic data from PRJNA304223. It was reported that ethylene is involved in nitrate-dependent root growth and branching in *Arabidopsis thaliana* (Tian et al., 2009). These results indicated that it is possible that the ethylene synthesis pathway contributes to nitrate signaling in maize.

**Table 1:** Transcriptional responses to nitrate of genes in the leading-edge subset of the ethylene action pathway in GSEA.

Gene ID	Log2FldChange	P-value	Description
*Zm00001d011208*	3.64	3.94E-88	1-aminocyclopropane-1-carboxylate (ACC) oxidase
*Zm00001d026060*	1.91	1.82E-11	1-aminocyclopropane-1-carboxylate (ACC) synthase
*Zm00001d025050*	0.76	6.27E-14	ETR/ERS-type ethylene receptor protein
*Zm00001d033862*	2.13	4.17E-07	1-aminocyclopropane-1-carboxylate (ACC) synthase
*Zm00001d039487*	1.15	0.010447	1-aminocyclopropane-1-carboxylate (ACC) synthase
*Zm00001d015778*	0.51	8.14E-05	EBF-type ethylene signal transducer
*Zm00001d000163*	0.82	0.048724	1-aminocyclopropane-1-carboxylate (ACC) oxidase
*Zm00001d003451*	0.79		EIN3-type ethylene signal transducer
*Zm00001d002592*	0.65	0.143665	1-aminocyclopropane-1-carboxylate (ACC) synthase
*Zm00001d007188*	0.47	0.354825	EIN3-type ethylene signal transducer
*Zm00001d013492*	0.53	0.306904	EIN2-type ethylene signal transducer
*Zm00001d024843*	0.26		1-aminocyclopropane-1-carboxylate (ACC) oxidase
*Zm00001d020686*	0.86	0.084843	1-aminocyclopropane-1-carboxylate (ACC) oxidase
*Zm00001d022530*	0.44		EIN3-type ethylene signal transducer transcript protein)
*Zm00001d024853*	0.45		1-aminocyclopropane-1-carboxylate (ACC) oxidase
*Zm00001d031445*	0.34	0.035294	EIN3-type ethylene signal transducer
*Zm00001d051889*	0.24	0.337379	ETR/ERS-type ethylene receptor protein
*Zm00001d036880*	0.30	0.427674	EBF-type ethylene signal transducer
*Zm00001d039341*	0.22	0.271768	EIN2-type ethylene signal transducer
*Zm00001d018211*	0.39	0.412937	1-aminocyclopropane-1-carboxylate (ACC) oxidase

Transcriptions of genes were determined based on Salmon-SMEM transcript quantification using the clean data derived from PRJNA283053. Genes expression changes were analyzed by using the R package DESeq2.

### 3.2 *ACO* and *ACS* gene families in maize

Given that ACO and ACS proteins were the only two enzymes responsible for ethylene biosynthesis, we aimed to identify the master *ACO* and *ACS* genes involved in root response to nitrate. First, we retrieved all *ACO* and *ACS* candidate genes in maize B73 genome through RBH search using ACO and ACS protein sequences of *Arabidopsis* as queries. ACOs were encoded by five genes in *Arabidopsis* genome, including *AtACO1* (*AT2G19590.1*), *AtACO2* (*AT1G62380.1*), *AtACO3* (*AT1G12010.1*), *AtACO4* (*AT1G05010.1*), and *AtACO5* (*AT1G77330.1*) (Houben and Van de Poel, 2019), while 16 homologs were found in maize genome (**Table 2**), of which *Zm00001d004718* and *Zm00001d004719* have the same sequences and were named as the same gene *ACCO6* in the database, and *Zm00001d024850* and *Zm00001d024851* were named as the same gene *ACCO4* (https://maizegdb.org/, accessed on March 11, 2025).

**Table 2:** ACO homologs from maize B73 genome.

Proteins ID	Gene ID	Gene Name
Zm00001d036955_P001	*Zm00001d036955*	*acco1*
Zm00001d020686_P001	*Zm00001d020686*	*acco2*
Zm00001d024852_P001	*Zm00001d024852*	*acco3*
Zm00001d024850_P001	*Zm00001d024850*	*acco4*
Zm00001d024851_P001	*Zm00001d024851*	*acco4*
Zm00001d011208_P001	*Zm00001d011208*	*acco5*
Zm00001d004718_P001	*Zm00001d004718*	*acco6*
Zm00001d004719_P001	*Zm00001d004719*	*acco6*
Zm00001d000163_P001	*Zm00001d000163*	*acco7*
Zm00001d024853_P001	*Zm00001d024853*	*acco15*
Zm00001d052136_P001	*Zm00001d052136*	*acco20*
Zm00001d024843_P001	*Zm00001d024843*	*acco31*
Zm00001d018211_P001	*Zm00001d018211*	*acco35*
Zm00001d005927_P001	*Zm00001d005927*	
Zm00001d015860_P001	*Zm00001d015860*	
Zm00001d046848_P001	*Zm00001d046848*	

Eight authentic *ACS* genes including *AtACS2* (*AT1G01480*), *AtACS4* (*AT2G22810*), *AtACS5* (*AT5G65800*), *AtACS6* (*AT4G11280*), *AtACS7* (*AT4G26200*), *AtACS8* (*AT4G37770*), *AtACS9* (*AT3G49700*), and *AtACS11* (*AT4G08040*) were reported in *Arabidopsis* (Yamagami et al., 2003). It was interesting that only four *ACS* homologs were identified in the B73 genome, which included *ZmACS1*, *ZmACS2*, *ZmACS6*, and *ZmACS7*. *ZmACS3* was not identified in this research, but it was also annotated as *ACS* in the database (https://maizegdb.org/, accessed on March 11, 2025) (**Table 3**). These homologs were subjected to further analyses except that *ZmACS3* was not included in the phylogenetic tree analyses.

**Table 3:** ACS homologs from maize B73 genome.

Proteins ID	Gene ID	Gene Name
Zm00001d039487_P001	*Zm00001d039487*	*acs1*
Zm00001d002592_P001	*Zm00001d002592*	*acs2*
Zm00001d033862_P001	*Zm00001d033862*	*acs6*
Zm00001d026060_P002	*Zm00001d026060*	*acs7*
–	*Zm00001d045479*	*acs3*

**Figure 2:**
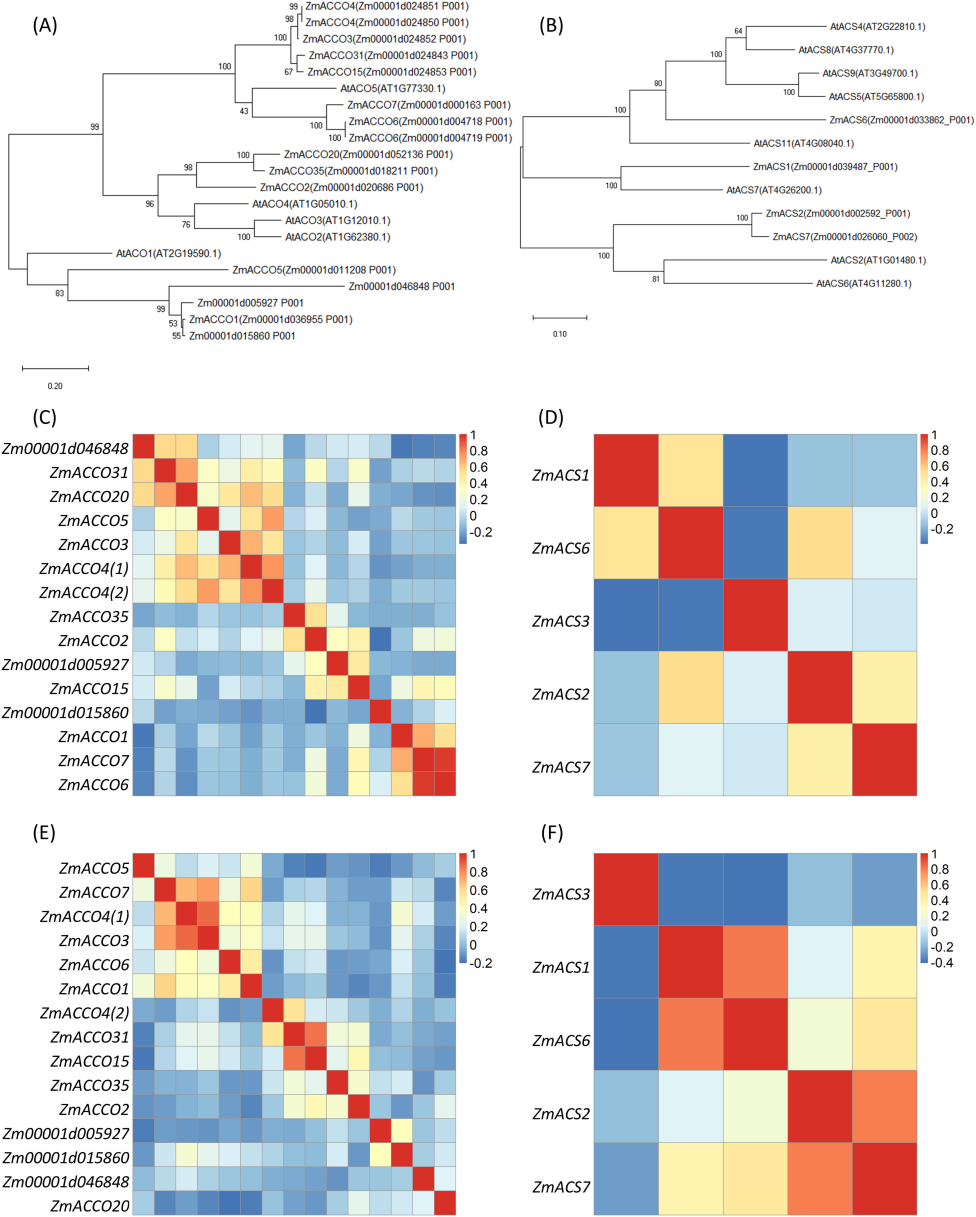
Relationships between Maize ACOs and between ACSs. **(A)** A phylogenetic tree of ACO proteins of maize and *Arabidopsis*. **(B)** A phylogenetic tree of ACS proteins of maize and *Arabidopsis*. **(C, D)** Expression correlations between *ACO* **(C)** and *ACS* **(D)** genes calculated with data from PRJNA2170531. **(E, F)** Expression correlations between *ACO* **(E)** and *ACS* **(F)** genes calculated with data from PRJNA171684. Maize ACO and ACS homologs were obtained by search maize database with known *Arabidopsis* ACO and ACS proteins using RBH method. Protein sequences were aligned by ClustalW and phylogenetic trees were constructed with maximum likelihood method. Transcriptions of genes were determined based on Salmon-SMEM transcript quantification using the clean data. The colors in **(C-F)** indicates the values of correlation coefficients. *ZmACCO4(1)* and *ZmACCO4(2)* are corresponding to *Zm00001d024850* and *Zm00001d024851*, respectively.

We next inspected the evolution relationships and expression correlations between these *ACO*s and *ACS*s from maize. The phylogenetic tree showed that most of these genes might result from duplicate events after the divergence between the ancestors of maize and *Arabidopsis*. In these processes, more *ACO* genes were kept in the maize genome, while more *ACS* genes were kept in the *Arabidopsis* genome (**Figures 2A,B**). As expected, there were no evolution distance between *ZmACCO4* (*Zm00001d024850*) and *ZmACCO4* (*Zm00001d024851*) and between *ZmACCO6* (*Zm00001d004718*) and *ZmACCO6* (*Zm00001d004719*). It was observed that the relationships between *ZmACCO3*, *ZmACCO4*, *ZmACCO15*, and *ZmACCO31* and between *ZmACCO1* and *Zm000015860* were close in the evolution. The *ACS* genes were shown to be more divergent compared with *ACO* genes in maize, and *ZmACS2* and *ZmACS7* are closer to each other in the evolution among all of the *ACS* genes.

The expression correlations between *ACO* genes and that between *ACS* genes were investigated with RNA-Seq data from PRJNA217503 (Walley et al., 2016) and PRJNA171684 (Stelpflug et al., 2016), respectively. These two projects included profiling data of gene expression in multiple tissues of B73. *ZmACCO6* (*Zm00001d004719*) was not included in the analysis as it has the same sequence as *ZmACCO6* (*Zm00001d004718*). Data from PRJNA217503 demonstrated that similar expression patterns existed between *ZmACCO6* (*Zm00001d004718*), *ZmACCO7*, and *ZmACCO1*, between *ZmACCO20*, *ZmACCO31*, and *Zm00001d046848*, between *ZmACCO4(1)* (*Zm00001d024850*), *ZmACCO4(2)* (*Zm00001d0024851*), *ZmACCO3*, and Z*mACCO5* in *ACO* genes (**Figure 2C**; **Supplementary Figure S1A**). A high expression correlation between *ZmACCO3* and *ZmACCO4(1)* (*Zm00001d024850*) was also observed in the data from PRJNA171684 (**Figure 2E**; **Supplementary Figure S1C**). The high expression correlations between *ZmACCO6* (*Zm00001d004718*) and *ZmACCO7* and between *ZmACCO4(1)* (*Zm00001d0024850*), *ZmACCO4(2)* (*Zm00001d0024851*) and *ZmACCO3* were consistent with their evolutional relationships observed in the phylogenetic tree (**Figure 2A**). The high expression correlation between *ZmACCO15* and *ZmACCO31* only observed in the data from PRJNA171684 (**Figure 2E**; **Supplementary Figure S1C**) was also consistent with their evolutional relationship (**Figure 2A**). On the whole, no clear links between the expression correlations and the evolutionary distances between these genes were observed.

For *ACS* genes, both data sets supported the distinctive expression pattern of *ZmACS3* compared with the other four genes (**Figures 2D, F**; **Supplementary Figures S1B,D**), which was in line with the result that *ZmACS3* was not identified in our homolog search. Relatively high expression correlations between *ZmACS1* and *ZmACS6* and between *ZmACS2* and *ZmACS7* were observed in both data sets (**Figures 2D, F**; **Supplementary Figures S1B, D**). The latter was consistent with their evolutionary relationship (**Figure 2B**).

**Figure 3:**
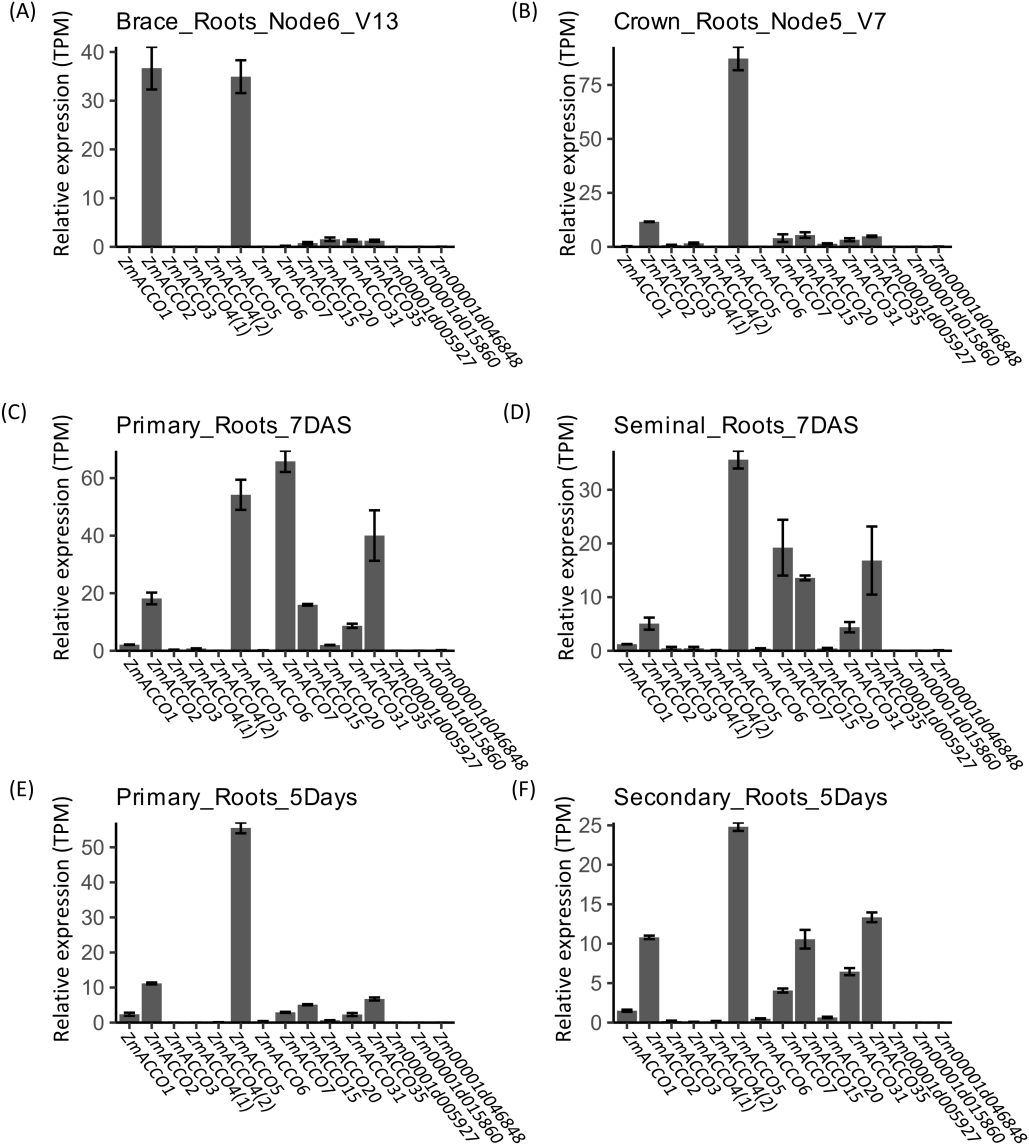
*In silico* assay of transcription of *ACO* genes in different type of roots of B73. **(A–D)** Expression of *ACO* genes estimated with the data from PRJNA171684. **(E, F)** Expression of *ACO* genes estimated with the data from PRJNA217053. Relative gene expression was calculated based on results from Salmon-SMEM quantification of transcripts and indicated with TPM (Transcripts Per Kilobase Million). Data were means ± SE (n = 3). *ZmACCO4(1)* and *ZmACCO4(2)* are corresponding to *Zm00001d024850* and *Zm00001d024851*, respectively.

We further examined the mRNA levels of these *ACO* and *ACS* genes in maize roots in detail. Data from PRJNA171684 demonstrated that the transcription profiles of *ACO* genes in different types of roots were similar (**Figures 3A–D**). The expression profiles of *ACO* genes in brace roots showed a higher similarity to that of crown roots (**Figure 3A, B**), while that in primary roots exhibited a higher similarity to that in seminal roots (**Figure 3C, D**). Similar profiles of *ACO* genes were also observed in the data from PRJNA217503 (**Figures 3E, F**). A high relative expression of *ZmACCO2*, *ZmACCO5*, *ZmACCO7*, *ZmACCO15*, *ZmACCO31*, and *ZmACCO35* was observed in both primary roots (**Figure 3C**) and seminal roots (**Figure 3D**) data from PRJNA171684 which were also perceived in the seminal roots data (**Figure 3F**) from PRJNA217503. Each of the maize *ACO* gene clusters in the phylogenetic tree has at least one member being relatively highly expressed in roots except for the two clusters to which *ZmACCO1* and *Zm00001d046848* belong, respectively (**Figure 2A**). The much higher expression of *ZmACCO7* and *ZmACCO35* observed in the primary root data from PRJNA171684 was not well confirmed by the data from PRJNA217503 (**Figures 3C, E**). No expression of *Zm00001d005927* was detected in all types of root data from both projects.

**Figure 4:**
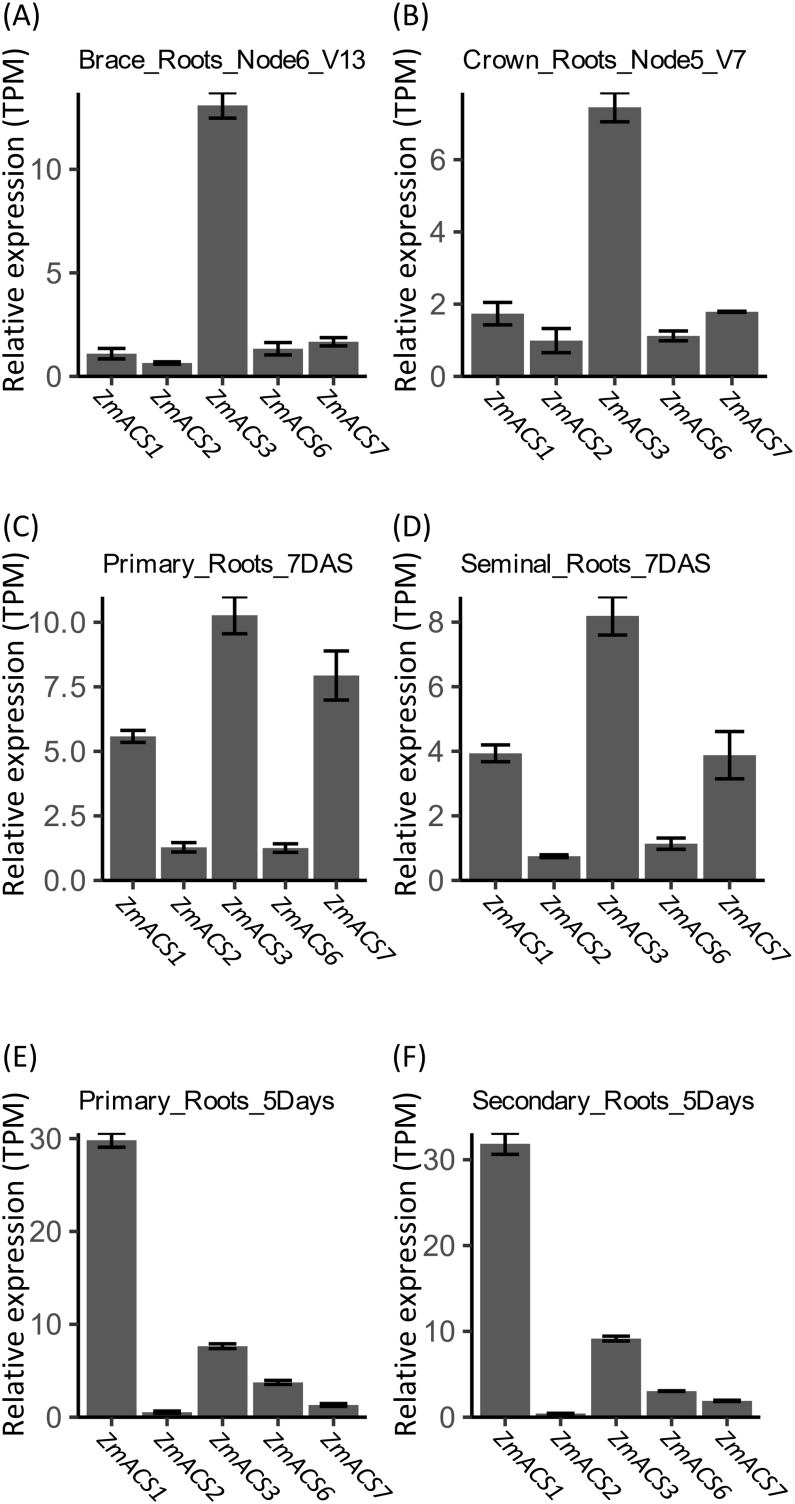
*In silico* assay of expression of *ACS* genes in different type of roots of B73. **(A–D)** Expression of *ACS* genes estimated with the data from PRJNA171684; **(E, F)** Expressions of *ACS* genes estimated with the data from PRJNA217053. Relative gene expression was calculated based on results from Salmon-SMEM quantification of transcripts and indicated with TPM (Transcripts Per Kilobase Million). Data were means ± SE (n = 3).

As observed for *ACO* genes, the expression profile of *ACS* genes in brace roots exhibited a higher similarity to that in crown roots (**Figures 4A, B**), while the expression profile of *ACS* genes in the primary roots showed a higher similarity to that in seminal roots (**Figures 4C–F**). However, differences were obvious between the expression profiles of *ACS* genes in primary roots or seminal roots from these two projects (**Figures 4C–F**). *ZmACS1*, *ZmACS3*, and *ZmACS7* were the top three highly expressed genes in both primary and seminal roots according to the data from PRJNA171684 (**Figures 4C, D**), each of which belongs to one of the three clusters of expression mentioned above (**Figures 2D, F**), while *ZmACS1* was the main expressed *ACS* gene based on the data from PRJNA217503 (**Figures 4E, F**).

Taken together, *ZmACCO2*, *ZmACCO5*, *ZmACCO7*, *ZmACCO15*, *ZmACCO31*, and *ZmACCO35* among *ACO* genes and *ZmACS1*, *ZmACS3*, and *ZmACS7* among the *ACS* genes in the ethylene synthesis pathway might be the master ACO or ACS genes function in maize roots.

### 3.3 Expression changes of *ACO* and *ACS* genes in response to nitrate

**Figure 5:**
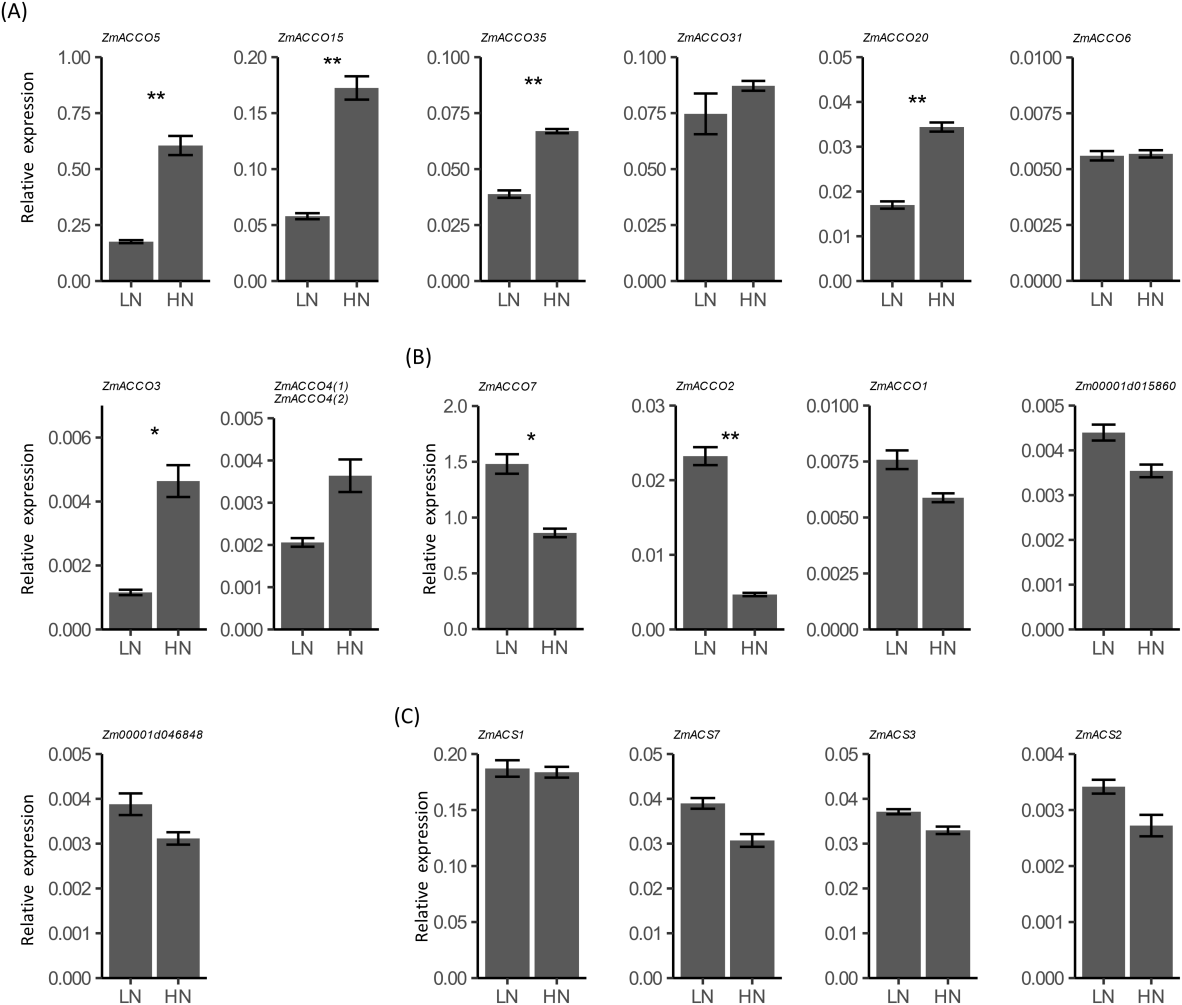
Relative mRNA expression of maize *ACO* and *ACS* homologs in response to nitrate provision. **(A)** Relative expression of *ACO* homologs of which expression is induced by nitrate in the roots of 6-day-old seedlings of B73 deficient in nitrate treated with 2.0 mmol/L Ca(NO3)_2_ (HN) or 2.0 mmol/L CaCl_2_ (as the mock treatment, LN) for 24 hours. **(B)** Relative e expression of *ACO* homologs of which expression is repressed by nitrate. **(C)** Relative expression of *ACS* homologs. Values are means ± SE (n = 7, 2 technical replicates were performed). ** and * indicate P < 0.01, P < 0.05 with Student’s t-test, respectively. *FPGS* was used as the reference gene. Expression of *ZmACCO4(1)* (*Zm00001d024850*) and *ZmACCO4(2)* (*Zm00001d024851*) was investigated with a common primer pair.

We examined the transcription responses of all of these *ACO* and *ACS* genes in the roots of B73 seedlings to nitrate provision with qPCR. Since no specific primers for *ZmACCO4(2)* (*Zm00001d024851*) were obtained, the total expression of *ZmACCO4(1)* (*Zm00001d024850*) and *ZmACCO4(2)* (*Zm00001d024851*) was investigated with a common primer pair. Amplification of the *ACO* homolog *Zm00001d005927* is not observed, and *ZmACCO5*, *ZmACCO15*, *ZmACCO35*, *ZmACCO31*, and *ZmACCO7* are highly expressed in the roots of B73 seedlings, which is consistent with the results of *in silico* analysis (**Figures 3**, **5A, B**). However, our data did not reveal a high expression of *ZmACCO2* relative to other *ACO* genes as *in silico* analysis demonstrated (**Figures 3C–F**, **5B**). The much high expression of *ZmACCO7* relative to other *ACO* genes observed in the data from PRJNA171684 is in line with our results (**Figures 3C, D**, **5B**). Some different from the results of the GSEA (**Table 1**), these *ACO* genes can be categorized into two groups according to their transcriptional responses to nitrate provision, with expression of genes in the first group being up-regulated by nitrate (**Figure 5A**) and that in the other group being down-regulated (**Figure 5B**). Expression of five genes was significantly up-regulated (**Figure 5A**) and expression of two was significantly down-regulated by nitrate provision (**Figure 5B**). Expression of all 7 the top high expressed *ACO* genes except *ZmACCO31*, which have relative expressions > 0.01 before treatment, was significantly regulated by nitrate provision (**Figures 5A, B**).

For *ACS* homologs, we failed to detect expression of *ZmACS6*. Results of qPCR showed that *ZmACS1* has much higher transcription relative to other *ACS* genes in roots of maize seedlings, which is in agreement with the result from the data of PRJNA217053 (**Figures 4E, F**, **5C**). Unlike *ACO* homologs, expression of all of the four *ACS* genes was slightly down-regulated by nitrate provision, and no significant regulations of these genes by nitrate were observed, which were also distinct from the results achieved from GSEA (**Figure 5C**; **Table 1**).

**Figure 6:**
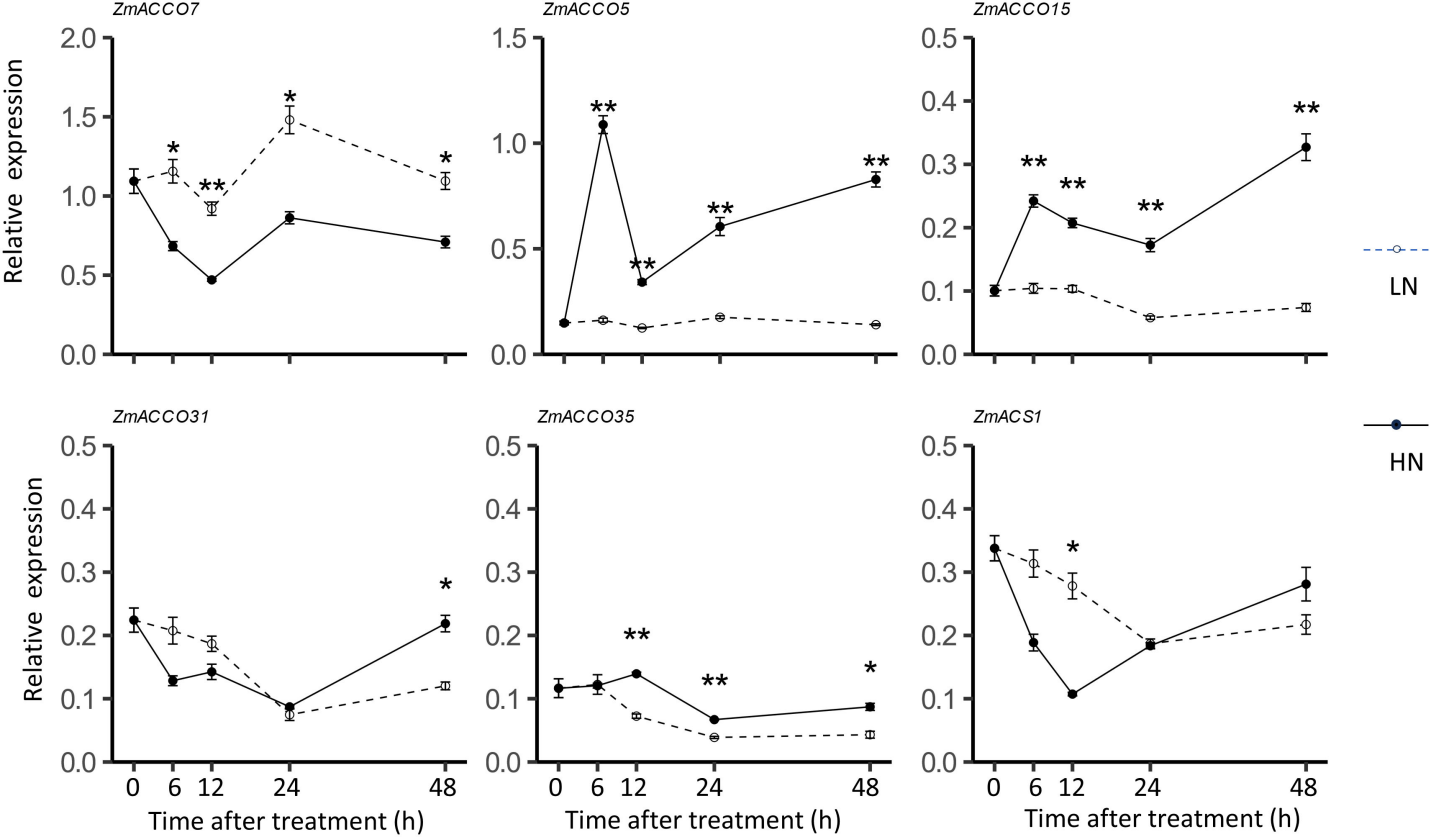
Dynamic mRNA expression of maize *ACO* and *ACS* homologs in response to nitrate provision. Relative mRNA levels of genes were detected in roots of 6-day-old B73 seedlings deficient in nitrate treated with 2.0 mmol/L Ca(NO3)_2_ (HN) or 2.0 mmol/L CaCl_2_ (as the mock treatment, LN). Values are means ± SE (n =6 or 7, 2 technical replicates were performed). ** and * indicate P < 0.01, P < 0.05 with Student’s t-test, respectively. *FPGS* was used as the reference gene.

To determine whether the time of treatments affected the detected regulatory modes of *ACO* and *ACS* genes in response to nitrate, we further examined the dynamic expression of the top five highly expressed *ACO* genes including *ZmACCO7*, *ZmACCO5*, *ZmACCO15*, *ZmACCO31*, and *ZmACCO35* as well as the most highly expressed *ACS* genes *ZmACS1* in response to the nitrate provision. The data of dynamic expression confirmed that four of the five *ACO* genes are induced by nitrate provision and that *ZmACCO7* is repressed (**Figure 6**). It was observed that regulations of these five genes by nitrate last longer than 2 days. No high extent of expression changes of *ZmACS1* in response to the nitrate treatment was observed at most time points (**Figure 6**), which was also in line with the results mentioned before.

Together, our experiment results and the *in silico* analyses suggested that *ZmACCO7*, *ZmACCO5*, *ZmACCO15*, *ZmACCO31*, and *ZmACCO35* are the top five highly expressed *ACO* genes in the roots of maize seedlings, and *ZmACS1* is the most highly expressed *ACS* gene. The expression of *ACO* and *ACS* genes is affected by nitrate provision in seedling roots of inbred line B73, but different regulatory modes exist for *ACO* genes and the expression response of *ACS* genes to nitrate provision is not obvious, which was not completely consistent with the results from GSEA.

### 3.4 Tissue-specific expression of *ACO* and *ACS* genes determines their particular regulatory modes in response to nitrate

**Figure 7:**
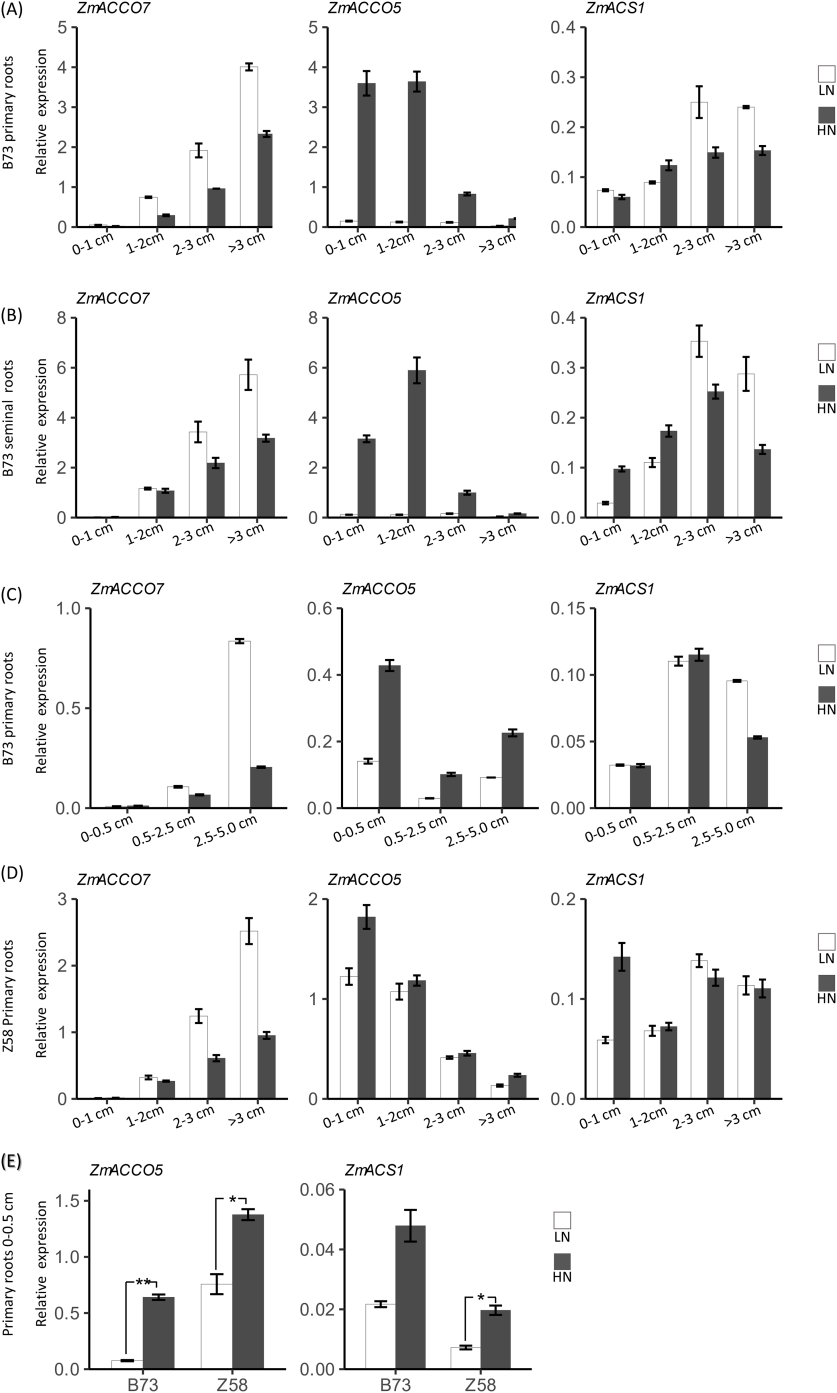
Root region-specific mRNA expression of maize *ACO* and *ACS* genes in response to nitrate provision. **(A)** Relative expression of *ZmACCO7*, *ZmACCO5* and *ZmACS1* in primary root segments of B73. **(B)** Relative expression of *ZmACCO7*, *ZmACCO5* and *ZmACS1* in seminal root segments of B73. **(C)** Relative expression of *ZmACCO7*, *ZmACCO5* and *ZmACS1* in primary root segments of B73. **(D)** Relative expression of *ZmACCO7*, *ZmACCO5* and *ZmACS1* in primary root segments of Z58. **(E)** Relative expression of *ZmACCO5* and *ZmACS1* in tips of primary roots of B73 and Z58. mRNA levels of genes were detected in roots of 6-day-old seedlings deficient in nitrate treated with 2.0 mmol/L Ca(NO3)_2_ (HN) or 2.0 mmol/L CaCl_2_ (as the mock treatment, LN) for 24 hours. For **(A–D)**, values are means ± SE, and 14 plants samples were bulked for RNA extraction and qPCR analysis. 3 technical replicates were performed. For **(E)**, values are means ± SE (n=6, 2 technical replicates were performed), and ** and * indicate P < 0.01, P < 0.05 with Student’s t-test, respectively. *FPGS* was used as the reference gene. The length in horizontal axes indicated start or end positions of sampled root segments relative to the root tip.

We speculated that the differences between regulatory modes of *ACO* and *ACS* genes in response to nitrate might be related to their tissue-specific expression. To test the hypothesis, we inspected the expression of three representative genes, *ZmACCO7*, *ZmACCO5*, and *ZmACS1*, in different segments of both primary roots and seminal roots of B73 in response to nitrate, including the root segments of 0–1 cm (Zone1), 1–2 cm (Zone2), 2–3 cm (Zone3), and >3 cm (Zone4) from root tips (**Figures 7A, B**). It was intriguing that *ZmACCO7* has a low expression in the root tips of primary roots, and its expression increases with distances from the root tip in primary roots while the expression pattern of *ZmACCO5* is opposite to that of *ZmACCO7* under both nitrogen conditions (**Figure 7A**). Similar results were obtained in seminal roots, except that the highest expression of *ZmACCO5* was detected in Zone2 root segments under high nitrogen condition (**Figure 7B**). *ZmACS1* has more uniform expression across root segments compared with the two *ACO* genes under both conditions (**Figures 7A, B**).

In agreement with the results mentioned before, the expression of *ZmACCO7* is repressed by nitrate provision, while the expression of *ZmACCO5* is induced (**Figures 7A, B**). These results indicated that the tissue-specific expression indeed determines their regulatory modes in response to nitrate. It was interesting that the expression of *ZmACS1* is induced by nitrate in root segments near the root tip and repressed in root segments far from the root tip, although its expression changes in response to nitrate provision are obviously lower than that of the two *ACO* genes (**Figures 7A, B**), which interpreted the result that no obvious expression response to nitrate provision was detected for *ZmACS1* in the samples of whole roots. The more uniform expression of *ZmACS1* across root segments might determine that its expression is upregulated in root regions near the root tip and downregulated in root regions far from the root tip by nitrate provision. In addition, it was also observed that the expression of *ZmACCO5* is extremely highly regulated by nitrate provision compared with *ZmACCO7*.

To confirm the results and inspect the effects of root regions on the regulations of *ACO* and *ACS* genes by nitrate in more detail, we examined the expression of these three genes in primary root segments sampled at positions with some differences from that we used above, i.e., 0–0.5 cm (Zone1), 0.5–2.5 cm (Zone2), and 2.5–5 cm (Zone3) (**Figure 7C**). As expected, the regulatory modes of *ZmACCO7* and *ZmACCO5* in response to nitrate in this experiment are similar to the results mentioned above (**Figure 7C**). *ZmACS1* still exhibits a more uniform expression across root segments and lower expression changes in response to nitrate provision in Zone1 and Zone2 segments (**Figure 7C**).

Taken together, *ACO* and *ACS* genes have different tissue-specific expression patterns across maize root regions which determine their particular regulatory modes in response to nitrate. *ACO* genes like *ZmACCO7* which are mainly expressed in root regions far from the root tip and are repressed by nitrate, while those like *ZmACCO5* which are mainly expressed in root regions close to the root tip are induced. *ACS* genes have relatively uniform expression across root regions and are induced in root regions close to the root tip but repressed by nitrate in root regions far from the root tip. As a result, nitrate provision might promote the production of ethylene in regions near the root tip but repress the production of ethylene in regions far from the root tip through the regulation of *ACO* and *ACS* genes at mRNA levels.

### 3.5 Differences in the expression responses of *ACO* and *ACS* genes to nitrate exist between genotypes

To know whether different genotypes share similar regulatory modes of expression of *ACO* and *ACS* genes in response to nitrate in roots, we next explored the mRNA expression of the three representative genes in response to nitrate provision in the primary roots of another maize inbred line Z58. The relative expression levels of the three genes in Z58 were comparable to that in B73, and their expression patterns across the primary root segments in Z58 were also similar to that in B73 (**Figures 7A, D**). The regulatory modes of these three genes in response to nitrate in Z58 were likewise similar to that in B73. However, obvious differences in expression change extents for both *ZmACCO5* and *ZmACS1* existed between the two genotypes (**Figures 7A, D**). The repression extent of *ZmACCO7* by nitrate in B73 was comparable to that in Z58, but the extreme induction of *ZmACCO5* by nitrate in B73 was not observed in Z58. The expression of *ZmACCO5* under the conditions of nitrate provision was 24.0, 29.0, 7.1, and 7.0 folds of that under the mock treatment in the zone1 to zone4 of B73 seedling primary roots, respectively (**Figure 7A**), while its expression under the conditions of nitrate provision was 1.5, 1.1, 1.1, and 1.8 folds of that under the mock treatment in the four corresponding root segments of Z58 plants, respectively (**Figure 7D**). Interestingly, induction of the *ZmACS1* by nitrate in Zone1 of Z58 roots (with a HN/LN expression ratio of 2.4) was stronger than that observed in B73 roots (**Figures 7A, D**). To confirm the differences in regulations of *ZmACCO5* and *ZmACS1* by nitrate between the two genotypes, we reexamined their expression responses to nitrate provision in the root tips of these two lines with an independent experiment. It was demonstrated that the expression of *ZmACCO5* under the nitrate treatment was 8.5 and 1.8 folds of that under the mock treatment in B73 and Z58, respectively (**Figure 7E**). Although the induction of *ZmACS1* by nitrate was more obvious in B73 compared with that observed before, it was still lower than that detected in Z58 (**Figure 7E**).

These data suggested that differences exist between genotypes in the regulation of *ACO* and *ACS* genes by nitrate. We suspect that it might be the defect in the transcriptional regulation of *ACS* gene(s) by nitrate in the regions near the root tip in B73 that results in the extreme induction of *ACO* gene(s) by nitrate due to a feedback effect as ACSs function upstream of ACOs.

### 3.6 Relationship between ethylene synthesis and nitrate-dependent root growth

**Figure 8:**
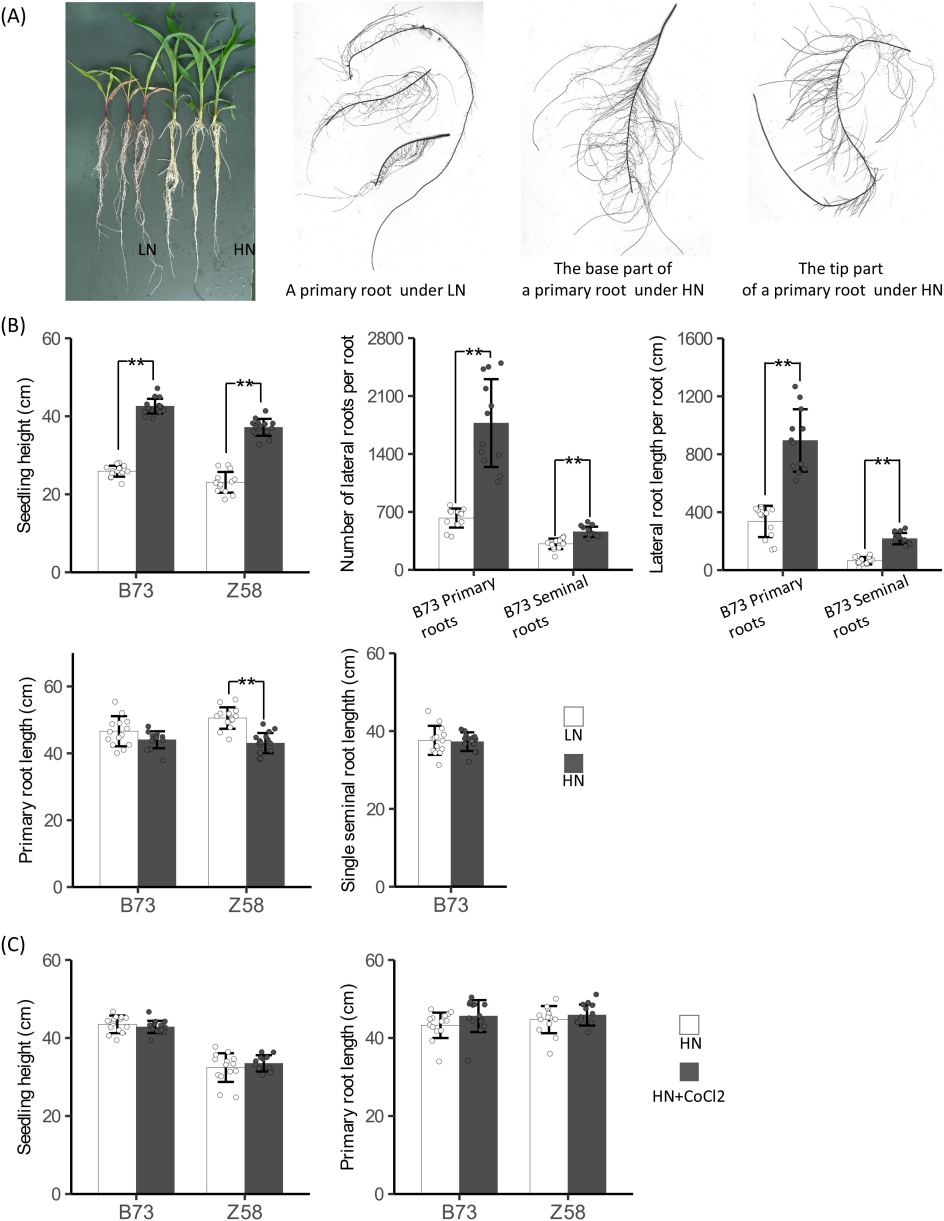
Relationships between ethylene synthesis and root growth. **(A)** Seedling growth of B73 under low nitrogen condition (LN) and high nitrogen condition (HN). **(B)** Quantitative analysis of seedling height and root growth of B73 and Z58 plants under LN and HN. **(C)** Quantitative analysis of seedling height and primary root length of B73 and Z58 plants grown under HN and HN + 3 µmol/L CoCl_2_. Five-day-old seedlings were subjected to hydroponic culture with 2.0 mmol/L Ca(NO3)_2_ (HN) or 2.0 mmol/L CaCl_2_ (LN) or 2.0 mmol/L Ca(NO3)_2_ plus 3 µmol/L CoCl_2_ (HN + CoCl2) for 9 days. For **(B)** and **(C)**, values are means ± SD (n ≥ 14 for seedling height; n ≥ 12 for primary related traits; n ≥ 14 for seminal roots related traits), and ** indicates P < 0.01 with Student’s t-test, respectively.

To reveal the role of ethylene synthesis in nitrogen-regulated plant growth in maize, we investigated B73 seedling growth under hydroponic conditions containing different concentrations of nitrate. As expected, plants with supply of 4 mmol/L nitrate were stronger and had more abundant biomass than that without nitrate supply after 9 days of treatments (**Figures 8A, B**). The total number of lateral roots and the total length of lateral roots per root of plants with nitrate supply were also significantly higher than that of plants without nitrate supply for both primary and seminal roots (**Figure 8B**). The observed nitrate-promoted lateral root growth is in line with other reports in maize (Gao et al., 2015) but different from the results from *Arabidopsis* where a high concentration of nitrate inhibits lateral root growth (Tian et al., 2009). However, the length of primary roots and seminal roots of B73 plants with nitrate supply was shorter than that of plants without nitrate supply (**Figures 8A, B**), which is in agreement with both the results from maize (Chun et al., 2005; Tian et al., 2008) and *Arabidopsis* (Linkohr et al., 2002; Ma et al., 2014) although the difference was not significant. These data indicated that maize has a specific regulatory mode of root growth in response to nitrate that high nitrate represses the elongation of primary and seminal roots but stimulates the growth of lateral roots on axial roots. High ethylene generally represses root growth (Khan et al., 2015). In *Arabidopsis*, high nitrate promotes ethylene synthesis to repress lateral root growth by upregulating the expression of *ACO* and *ACS* genes (Tian et al., 2009). The regulatory mode of root growth in response to nitrate in maize is also consistent with the regulations of *ACO* and *ACS* genes by nitrate that high nitrate upregulates the mRNA levels of *ACO* and *ACS* genes on the whole in root regions near the root tip of axial roots but downregulates their mRNA levels in the root regions far from the root tip.

To know whether the differences in regulations of *ACO* and *ACS* genes by nitrate between genotypes are associated with their root growth responses to nitrate provision, we next investigated the growth responses of Z58 seedlings to nitrate under hydroponic conditions. High nitrate stimulated shoot growth and repressed the primary root growth in Z58 as observed in B73 (**Figure 8B**). However, the primary roots of Z58 exhibited a higher extent of response to nitrate provision compared with that of B73 (**Figure 8B**). Z58 plants with nitrate supply demonstrated 8.5% reduction of primary root length compared to the control, while B73 plants with nitrate supply exhibited 5.5% reduction relative to the control (**Figure 8B**). These results might indicate that the defect in regulation of *ZmACS1* by nitrate in B73 is linked to its weaker primary root growth responses to nitrate where less effective regulation of *ACS* genes by nitrate might result in less ethylene production, although *ACO* genes are extremely induced by nitrate as ACSs act upstream ACOs in the ethylene synthesis pathway. To test the hypothesis that high nitrate inhibits elongation of primary and seminal roots in maize through upregulating *ACO* and *ACS* genes to elevate the production of ethylene, we examined root growth under a condition containing both the ethylene synthesis repressor CoCl_2_, an antagonist for ACO (Lau and Yang, 1976), and nitrate. As expected, 3 µmol/L CoCl_2_ relieved the inhibition of primary root growth of both B73 and Z58 by nitrate, although the effects were not significant (**Figure 8C**).

## 4 Discussion

In this research, we found that the ethylene synthesis pathway is evolved in nitrate signaling in maize seedling roots and participates in nitrate-dependent root growth. Moreover, we observed that there exist differences in the transcriptional regulations of *ACO* and *ACS* genes by nitrate between genotypes.

A wealth of information is contained in the size-increasing public next-generation sequencing (NGS) database. GSEA is a robust approach to identify valuable clues linked to a defined gene set in these data (Subramanian et al., 2005). However, in this study, we found enrichment of the ethylene action pathway in only one of the two explored transcriptomic data. Our further experiments showed that members of the same gene family have different regulatory modes in different tissues. These results indicate that it is more possible to identify interesting results using data of a particular type of tissues or cells in NGS data mining with methods like GSEA.

The expression of genes in the ethylene synthesis pathway was upregulated by nitrate (10 mmol/L NO_3_^-^) in *Arabidopsis* (Tian et al., 2009). We found that, in maize seedlings, the expression of *ACO* and *ACS* genes on the whole is stimulated in root regions close to the root tip but repressed in root regions far from the root tip by nitrate provision (4 mmol/L NO_3_^-^). These results indicated that maize has a nitrate regulatory mode for the ethylene synthesis pathway different from that of *Arabidopsis*. However, the relationships of ethylene synthesis pathway with root growth might be similar between species, i.e., upregulation of the ethylene synthesis pathway by nitrate is accompanied by inhibited root growth, while its downregulation promotes root growth, which agrees with the general view that ethylene is a stress hormone (Le et al., 2001; Swarup et al., 2007; Wang et al., 2002). Thus, it is possible to remodel the regulatory modes of particular members of gene families in the ethylene synthesis pathway in response to nitrate to obtain an ideotype of root architecture, such as to relieve the induction of *ZmACCO5* by nitrate in regions near the root tip to promote axial root growth in order to gain a deep rooting genotype for enhancing NUE. In this research, we only investigated relationships between root growth and transcription regulations of *ACO* and *ACS* genes by nitrate. Further works are required to unravel the underlying mechanisms. Additionally, how ethylene affects nitrate transport and metabolism in maize roots remains to be investigated.

We found that *ZmACCO7*, *ZmACCO5*, *ZmACCO15*, *ZmACCO31*, and *ZmACCO35* are the top five highly expressed *ACO* genes, and *ZmACS1* is the most highly expressed *ACS* gene in the roots of maize seedlings, which is consistent with some results of *in silico* analysis (**Figures 3**-**5**). However, an *in silico* expression analysis demonstrated that the master *ACO* and *ACS* genes in brace roots and crown roots might not be master genes in primary and seminal roots. Our analysis also exhibited tissue-specific expression of *ACO* and *ACS* genes in same roots. Crown roots and brace roots are most important root systems for maize during vegetative growth and reproductive development (Lynch, 2013). Therefore, it is necessary to confirm which members of this gene family are master *ACO* and *ACS* genes and investigate their regulatory modes in response to nitrate in crown roots and brace roots. In fact, data from the project of PRJNA283053 showed that *ZmACCO7* was upregulated by nitrate in brace roots, which is different from the result observed in this research.

Our analyses showed differences in the regulations of *ACO* and *ACS* genes existing between B73 and Z58 which might be associated with the differences in remodeling of root architectures by the nitrate between the two genotypes, i.e., weaker induction of *ZmACS1* in root regions near the root tips by nitrate might result in less reduction of primary root elongation by nitrate in B73 compared with Z58. Further experiments are needed to find the factors determining the regulatory differences in response to nitrate between genotypes and, moreover, to identify more diversity of regulation of *ACO* and *ACS* genes by nitrate in maize germplasm and to explore their application in improving NUE in maize.

In conclusion, we found that the ethylene synthesis pathway is involved in the responses to nitrate of maize seedling roots in a more complex mode relative to that in *Arabidopsis*, which is associated with remodeling of the root architecture by nitrate, and there exist differences in regulations of *ACO* and *ACS* genes between genotypes, which might be valuable in improving the NUE of maize varieties.

## Data Availability

The original contributions presented in the study are included in the article/**Supplementary Material**. Further inquiries can be directed to the corresponding author.
