# Supplementary figures and images for: Transcriptions of ACO and ACS genes are involved in nitrate-dependent root growth of maize seedlings
Source: Front Plant Sci. 2025 May 2;16:1566213. doi: 10.3389/fpls.2025.1566213 (PMC12081380; doi:10.3389/fpls.2025.1566213)

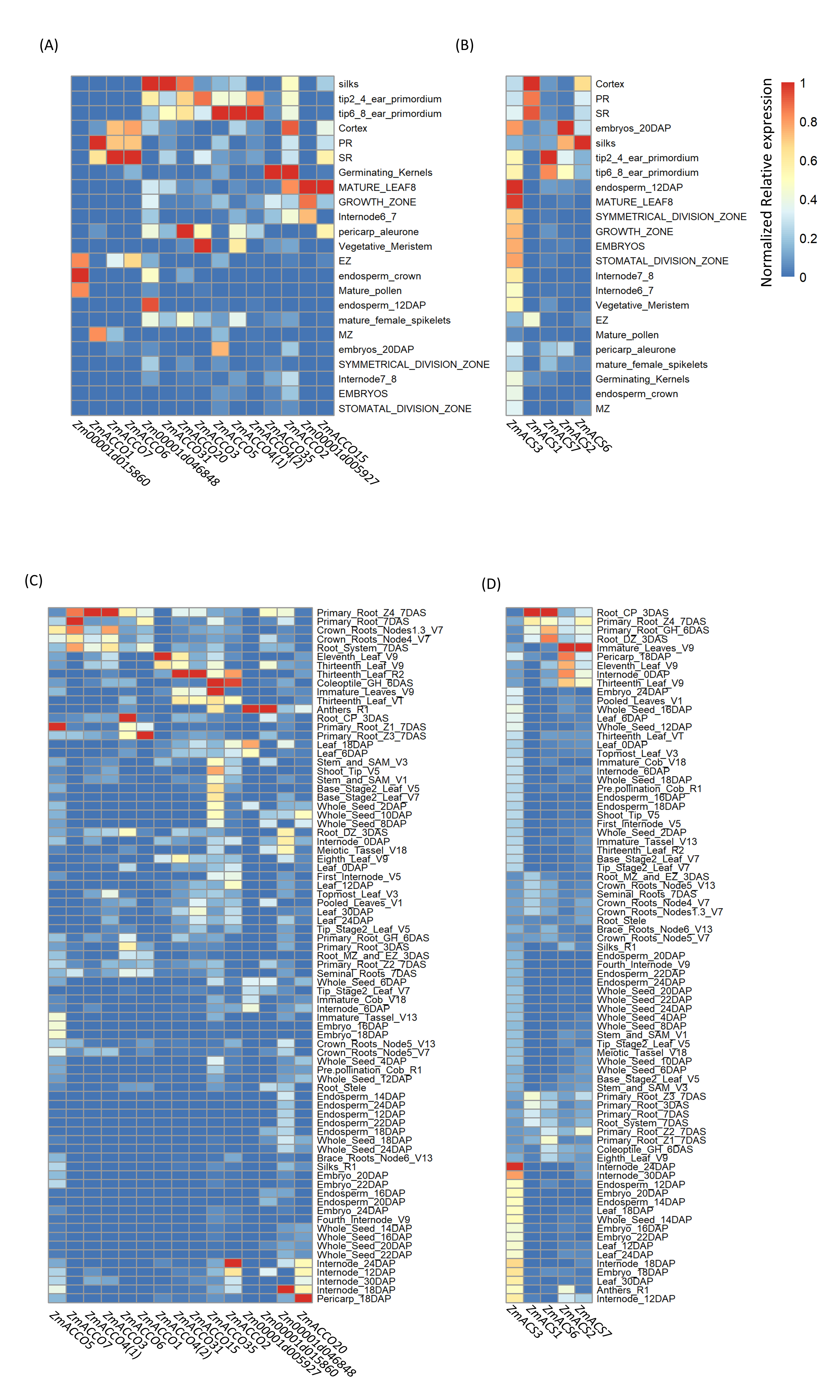

Supplement: Supplementary file 1 [file Image1.tif]
