# Supplementary material for: Transcriptions of ACO and ACS genes are involved in nitrate-dependent root growth of maize seedlings
Source: Front Plant Sci. 2025 May 2;16:1566213. doi: 10.3389/fpls.2025.1566213 (PMC12081380; doi:10.3389/fpls.2025.1566213)
Supplement: Supplementary file 2 [file Table1.docx]

Supplementary Material

# Supplementary Figures

Figure S1. Expression patterns of ACO and ACS genes. (A - B) Expression patterns of *ACO* genes (A) and *ACS* genes (B) based on data from PRJNA2170531. (C - D) Expression patterns of *ACO* genes(C) and maize *ACS* genes (D) based on data from PRJNA171684. Relative expression was presented. Normalized Relative expression of a gene in a tissue was calculated by dividing the TPM (Transcripts Per Kilobase Million) by the maximum TPM of a gene across all tissues.

# Supplementary tables

## Supplementary Data Sheet 1

Table S1. Primers used in this research.

## Supplementary Data Sheet 1

Table S2. Results of GSEA of transcriptome of maize in response to nitrate treatment.
